# Exosomal microRNAs in regulation of tumor cells resistance to apoptosis

**DOI:** 10.1016/j.bbrep.2024.101644

**Published:** 2024-01-17

**Authors:** Mohammad Salehi, Mohammad Javad Kamali, Daniyal Arab, Naghme Safaeian, Zahra Ashuori, Moein Maddahi, Narges Latifi, Amir Moein Jahromi

**Affiliations:** a Department of Medical Genetics, School of Advanced Technologies in Medicine, Golestan University of Medical Sciences, Gorgan, Iran; b Student Research Committee, Golestan University of Medical Sciences, Gorgan, Iran; c Department of Medical Genetics, School of Medicine, Babol University of Medical Sciences, Babol, Iran; d Department of Human Genetics, Science and Research Branch, Islamic Azad University, Tehran, Iran; e Tehran Medical Sciences, Islamic Azad University, Tehran, Iran; f Cellular and Molecular Biology Research Center, Health Research Institute, Babol University of Medical Sciences, Babol, Iran; g Faculty of Dentistry, Yeditepe University, Istanbul, Turkey; h Department of Cell and Molecular Biology & Microbiology, Faculty of Biological Science and Technology, University of Isfahan, Iran; i School of Dentistry, Tehran University of Medical Sciences, Tehran, Iran

**Keywords:** Apoptosis, Cancer, MicroRNA, Exosome, Drug resistance

## Abstract

Exosomes are a type of extracellular vesicle that contains bioactive molecules that can be secreted by most cells. Nevertheless, the content of these cells differs depending on the cell from which they originate. The exosome plays a crucial role in modulating intercellular communication by conveying molecular messages to neighboring or distant cells. Cancer-derived exosomes can transfer several types of molecules into the tumor microenvironment, including high levels of microRNA (miRNA). These miRNAs significantly affect cell proliferation, angiogenesis, apoptosis resistance, metastasis, and immune evasion. Increasing evidence indicates that exosomal miRNAs (exomiRs) are crucial to regulating cancer resistance to apoptosis. In cancer cells, exomiRs orchestrate communication channels between them and their surrounding microenvironment, modulating gene expression and controlling apoptosis signaling pathways. This review presents an outline of present-day knowledge of the mechanisms that affect target cells and drive cancer resistance to apoptosis. Also, our study looks at the regulatory role of exomiRs in mediating intercellular communication between tumor cells and surrounding microenvironmental cells, specifically stromal and immune cells, to evade therapy-induced apoptosis.

## Highlights

• Exosomes modulate intercellular communication by conveying molecular messages to neighboring or distant cells.
• Exosomes harbor miRNAs that play a critical role in cell death signaling pathways and therapy response.
• Targeting exosomal microRNAs holds significant potential as a therapeutic strategy to overcome cancer resistance.

## 1 Introduction

Cancer is characterized by uncontrolled cell growth due to gene mutations or malfunctions in cell division and growth mechanisms [[1], [2], [3]]. In 2020, there were an estimated 19.3 million newly diagnosed cancer cases worldwide and nearly 10.0 million cancer-related deaths [4,5]. Dysregulated genes or cellular mechanisms play a significant role in cancer development, particularly oncogenes and tumor suppressor genes [6]. Several “tumor suppressor genes" are activated and deactivated to regulate cancer initiation and progression [6,7]. As well as genetic mutation, chromosomal abrasion or alteration, such as addition or deletion, and changes to the cell cycle are important factors contributing to variation in established genetic mechanisms [8].

Apoptosis, also known as programmed cell death, is critical to cell proliferation, growth rates, and cancer development. This process is controlled by an active and energy-dependent genetic mechanism [9]. Apoptosis is characterized by cell shrinkage, chromatin condensation, blebbing, and apoptotic bodies [10,11]. This process associated with the preservation of an intact cell membrane and the absence of any signs of inflammation [12]. Certain proapoptotic stimuli, such as endoplasmic reticulum stress and reactive oxygen species, trigger apoptosis by activating caspases, which induce irreversible cell changes [10]. Apoptosis helps achieve a balanced state, preventing inflammatory reactions and tissue damage while maintaining the internal environment and protecting the host [13]. Cancer cells have evolved diverse mechanisms to evade apoptosis, enabling uncontrolled proliferation and survival. One common strategy is the acquisition of genetic mutations or alterations that disrupt or modify essential components of the apoptotic pathway. Mutations in oncogenes and tumor suppressor genes, which play a crucial role in regulating apoptosis, can significantly impact the apoptotic response [14].

Recent research has identified a new class of non-coding RNAs called microRNAs (miRNAs), which have been identified as potential regulators of the apoptosis of cancer cells [15]. A miRNA is composed of 18–25 nucleotides and regulates gene expression after transcription through mRNA degradation or translation inhibition [16,17]. It is estimated that miRNAs constitute less than 5 % of the human genome and regulate approximately more than 60 % of the mRNA transcripts and one-third of the protein-coding genes by loading into Argonaute (AGO) protein, forming the active RNA-induced silencing complex (RISC). In RISC, miRNA recognizes and binds to a specific guide sequence in the target mRNA, usually found in the 3′-untranslated region (3′-UTR). This imperfect binding often results in translational repression or mRNA destabilization. In some cases, there is nearly complete complementarity, which results in mRNA degradation and cleavage [18,19]. It has been shown that these molecules can act as oncogenes or tumor suppressors in cancers [20,21]. Moreover, miRNAs regulate cell differentiation, proliferation, and apoptosis by interacting with cancer target genes [22]. Most mature miRNAs are located in the cytoplasm, binding to target mRNAs. Moreover, miRNAs are also found in the extracellular space through active secretion, passive release, or encapsulation in extracellular vesicles such as exosomes. Thus, these exosomes serve as messengers for the transfer of miRNAs between cells [23]. This type of miRNAs, known as exosomal-microRNAs (exo-miRNAs or exomiRs), are secreted by cancer cells and can be taken up by neighboring cells, thereby altering gene expression and cellular behavior, which includes altering target cell behavior related to invasion, tumor growth, gene expression, angiogenesis, and immune response [24]. In view of this interest, new therapeutic strategies targeting exo-miRNAs are being investigated for their impact on cancer apoptosis [25]. This review focuses on the role of exosomal miRNAs in regulating communication between cancer cells and neighboring non-cancerous cells, such as stromal cells and immune cells, to evade apoptosis. To the best of our knowledge, our study presents a thorough overview that is specifically dedicated to the role of exosomal miRNAs in the mechanisms governing the suppression of apoptosis and the development of apoptosis resistance in tumor cells.

## 2 Exosomes

### 2.1 Definition and function

Exosomes are small extracellular vesicles (EVs) ranging in diameter from 30 to 150 nm, with an average diameter of 100 nm. They are enclosed by lipid bilayer membranes and carry various biological materials [26]. The first discovery of exosomes in serum in 1983 led to the assumption that exosomes were only transporting cellular waste with relatively low potential for research [27]. The release of exosomes into the extracellular environment occurs when multivesicular bodies (MVBs) or late endosomes fuse with the plasma membrane. In cell communication, they play a crucial role as important mediators [[26], [27], [28], [29]]. Exosomes are released by various cell types in the human body and are found abundantly in a wide range of bodily fluids. These include blood, urine, saliva, bile, ascites, breast milk, cerebrospinal fluid, and even within the tissue matrix [26,30,31]. Exosomes are utilized for transferring a diverse range of cargoes, including proteins, metabolites, and nucleic acids (such as non-coding RNA, miRNAs, mRNA, and DNA fragments) [[32], [33], [34], [35]]. Exosomes function in many ways, depending on cell types, local or systemic physiology, or pathology, including the transfer of signal molecules between cells and the remodeling of the extracellular matrix [28]. Exosomes significantly impact multiple aspects of human health, encompassing immunity, cancer, tissue homeostasis, neurodegenerative diseases, and human development [31]. Exosomes derived from cancer cells possess the capability to stimulate the formation of new blood vessels, thereby facilitating the delivery of nutrients, oxygen, and waste removal, as well as contribute to the metabolic reprogramming of cancer cells to promote a favorable microenvironment that supports tumor growth and prevents apoptosis for their sustained proliferation. These properties make exosomes suitable therapeutic agents for treating a range of diseases. This includes cancer treatment, reprogramming the tumor microenvironment, and modulating the immune response [24,36].

### 2.2 Composition and biogenesis

**Fig. 1:**
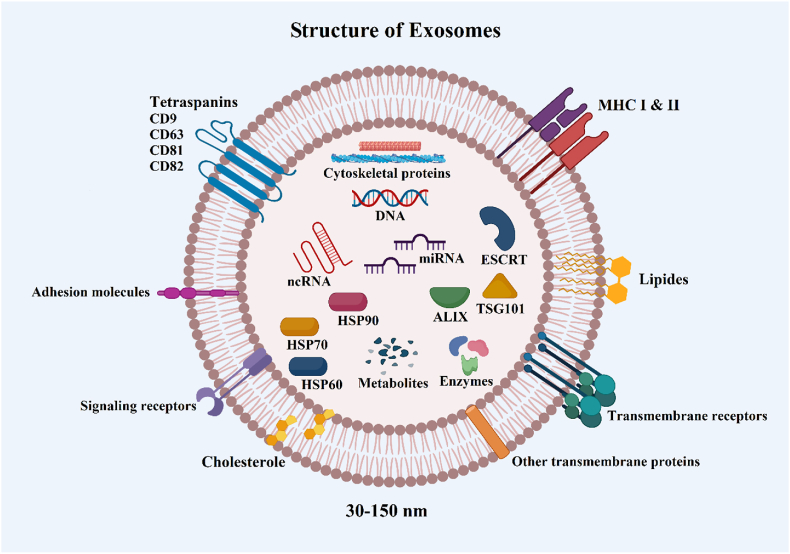
Exosomes, small extracellular vesicles, are characterized by a core set of markers, including CD9, CD63, CD81, CD82, and ALIX. These markers aid in their identification and isolation. Exosomes carry a diverse cargo of biomolecules, such as DNAs, mRNAs, ncRNAs, proteins, lipids, and metabolites. Cargo composition influences intercellular communication and cellular processes. Created with BioRender.com.

**Fig. 2:**
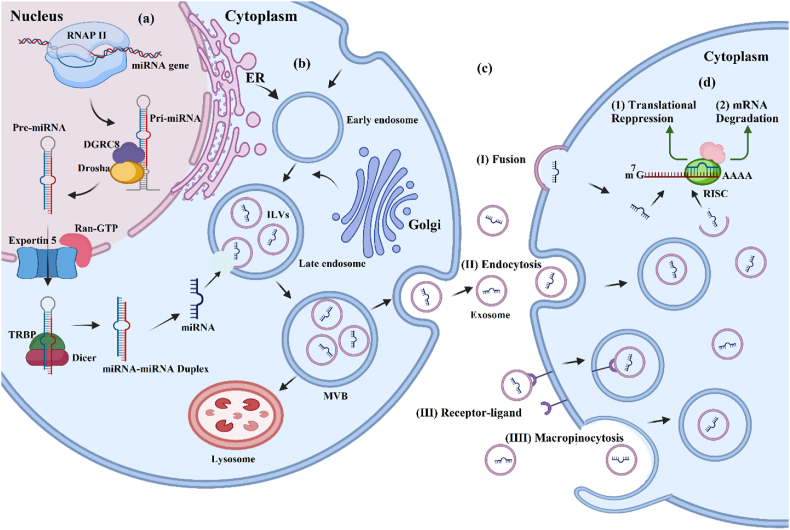
The sorting mechanism of exosomal-miRNAs. (a): This mechanism begins with the biogenesis of miRNAs in the nucleus. (b): In donor cell cytoplasm, the sorting of exomiRs into exosomes occurs. (c): exosomes are secreted from the donor cell and incorporated into the recipient cell by several mechanisms that have been shown. (d): Upon entering the recipient cell, exomiRs can bind to their target mRNAs' 3′-UTR (untranslated region). This binding leads to the degradation of the target mRNA or repression of translation, thereby limiting protein synthesis. Created with BioRender.com.

There is a common set of identical proteins present in exosomes of different cell types, including the tetraspanin family (CD9, CD63, CD81, and CD82), the endosomal sorting complex transport (ESCRT), heat shock proteins (HSP60, HSP70, HSP90), proteins of the molecular histocompatibility complex (MHC), Tsg101 and Alix (Fig. 1) [37]. Several steps are involved in exosome formation and secretion. Early endosomes are formed in the early stages of this process, which are small vesicles formed by plasma membrane budding inward [38]. The early endosome matures into the late endosome, a larger vesicle capable of fusing with similar late endosomes or lysosomes [38]. Proteins and lipids, assembled into intraluminal vesicles (ILVs), are sorted and packaged by ESCRT machinery within late endosomes. As a result, specific proteins and RNAs are selectively incorporated into the exosome, known as endosomal sorting [39]. Four proteins comprise the ESCRT machinery (ESCRT-0, –I, –II, and –III), which work in concert to identify cargo proteins and RNAs and sort them into ILVs [40]. Following the incorporation of the ILVs, the late endosome transforms into a multivesicular body (MVB) consisting of several ILVs. If the MVB is fused with the plasma membrane, its contents will be released into the extracellular space as exosomes, or if it is fused with a lysosome, the contents will be degraded [41]. The Rab27 small GTPase regulates the docking of MVBs at the membrane to release their contents as exosomes. Although the mechanism of exosome packing remains unknown, comparing the exosome composition with the parent cell indicates a selective enrichment process within the exosomes [42]. There are several mechanisms by which exosomes are incorporated into the recipient cell, including macro-pinocytosis, phagocytosis, endocytosis, and surface receptor interaction [43,44] (Fig. 2).

### 2.3 Detection and isolation

Exosomes are actively released from cells and can be isolated from various biofluids such as blood, urine, and saliva. They contain specific proteins, including tetraspanins (CD9, CD63, CD81, and CD82), Tsg101, and Alix. These proteins are involved in exosome biogenesis and serve as markers during isolation [44], along with the physical-chemical characteristics of exosomes, such as size, density, and buoyancy [45]. The International Society for Extracellular Vesicles (ISEVs) encourages the publication of methodological details to ensure reproducibility and reliability despite lacking a gold standard exosome isolation method [46]. There may be a reason for this, as it is difficult to isolate pure exosomes since body fluids contain soluble proteins as contaminants [47].

The most widely used method is ultracentrifugation, which spins a sample at high speeds to separate exosomes and other particles for long periods. In addition, size exclusion chromatography (SEC) is another method for separating particles based on their size, allowing exosomes to be isolated from other particles [48]. There are also polymer precipitation methods, immunoaffinity capture, which uses antibodies to specifically bind to exosomes, and microfluidics-based approaches, which use microchannels to separate particles according to their shape and size [49]. Significant advancements were made in 2018 and 2020 in developing more efficient methods for isolating high-quality exosomes, including techniques like asymmetric flow field-flow fractionation (AF4) and the integrated microfluidic chip (IMC) method [50,51]. In addition to large vesicles (90 nm–120 nm) and small vesicles (60 nm–80 nm), the AF4 protocol can separate nanoparticles of 35 nm in size. Conversely, IMC provides a platform for isolating exosome subsets based on surface markers, such as CD63. Apart from these two methods, an acoustic nano-filter system has also been developed, which allows vesicles to be separated continuously and contact-free [52].

## 3 Exosomal miRNAs biogenesis

Recent studies have increasingly focused on non-coding RNAs in biological medicine [53]. Exosomes transport several miRNAs to perform these biological tasks, and this complex is referred to as exosomal-miRNAs (exomiRs) [54]. The interaction between tumor cells through exomiRs leads to changes in cellular biological events. This influences the surrounding extracellular tumor matrix environment and ultimately contributes to drug resistance [55].

ExomiR sorting begins within the nucleus, and the following events occur in the cytoplasm. Initially, miRNAs are expressed by RNA polymerase II as primary-miRNA transcripts (Pri-miRNAs), which range in length from several hundred to several thousand nucleotides [56]. Using the DGCR8 and Drosha complex, pri-RNAs are converted into smaller stem-loop intermediate precursor-miRNAs (pre-miRNAs) of 80–100 nucleotides in length [57]. Exportin-5 (a dsRNA binding protein involved in a GTP-dependent transport process) recognizes and exports pre-miRNAs into the cytoplasm by recognizing and exporting them. RNAse Dicer, an endonuclease of RNase III-type, cleaves the stem structure at the extremities and releases a small RNA duplex [58]. The 5p and 3p segments of the fully processed double-stranded miRNA originate from the 5′ and 3′ ends of the pre-miRNA, respectively. Biological functions may differ between 5p and 3p due to their different mRNA-targeting properties [59]. After being loaded onto an Argonaute (AGO) protein, the RNA duplex is unwinded and forms an RNA-induced silencing complex (RISC) [60]. When a miRNA binds to a RISC, it pairs with the mRNA complementary. Two different mechanisms occur, depending on whether miRNA and mRNA are fully bound [1]: When miRNA is sufficiently complementary to mRNA, it specifies cleavage [2]; miRNAs that are not sufficiently complementary to mRNA inhibit productive translation [61,62]. As a result, miRNAs play a role in regulating various physiological and pathological processes.

## 4 Sorting mechanisms of exosomal miRNAs

Endosomal sorting allows ESCRT machinery to selectively incorporate proteins and RNAs into intraluminal vesicles (ILVs) to create exosomes, which can contain miRNAs. Consequently, miRNAs can be packaged into exosomes, which are released into the extracellular space for intercellular communication [63]. Exosomes may carry different kinds of miRNAs depending on the cell type, physiological state, and environmental environment. Several studies suggest that mRNAs and non-coding RNAs, such as miRNAs, enter exosomes as part of a specific sorting process [63]. It has been proposed that miRNAs contain intrinsic sorting signals necessary for their incorporation into exosomes [64]. In peripheral blood mononuclear cells, exosomes display one of those sorting mechanisms. An RNA binding protein, hnRNPA2B1, recognizes 4-bp RNA motifs, GGAG, if sufficiently sumoylated [65]. The same protein family, hnRNPC and hnRNPA1, can also bind to exomiRs. The associated motif has not yet been found, however. Exosomes from a CRC cell line (SW620) contained the RNA motif GUUG, similar to the GGAG motif that hnRNPA2B1 recognizes [66].

Secondly, there has been some evidence that the lipid composition of exosome membranes directly impacts the biogenesis and composition of exosomes; this is the case in several studies [[67], [68], [69]]. Also, this can affect the sorting of miRNAs into exosomes. For instance, neutral sphingomyelinase2 (nSMase2) is a regulator of ceramide synthesis and can affect the amount of miRNA exported by exosomes [70]. In colorectal cancer cells (CRCs) and hepatocellular carcinoma cells, sphingomyelin phosphodiesterase 3 (SMPD3) is also involved in miRNA encapsulation, and SMPD3 inhibition decreases exomiR levels in CRC cells while increasing intracellular miRNA levels [71]. The third hypothesis is that proteins play a role in miRNA biogenesis and function. This process of miRNA maturation is closely related to the export of miRNAs from exosomes and the endosomal trafficking of miRNAs. In several cell lines, Ago2 knockout significantly reduces specific miRNA populations [72]. Furthermore, RISC components (such as Dicer and Ago2) can co-localize with MVBs when the turnover of MVBs into lysosomes is blocked [73]. Exosomes with high levels of CD43 are suspected to be a mediator for active protein delivery to exosomes. In addition, this protein increases Dicer levels, a relationship between exosome processing and miRNA synthesis [74,75]. A study examined CRC cell-derived EVs and found passenger-strand (3p) miRNAs predominance over their 5p counterparts [76]. According to a mechanism identified in the cells, the KRAS small GTPase may play a role in miRNA sorting in CRC cells. More than a third of sporadic colorectal cancers have KRAS mutations, and they have also been linked to several other cancers, especially those with aggressive tumors [[77], [78], [79]]. It has been found that the ratio of miRNA in exosomes is more significant than in primary cells, even compared to other cargo carried by exosomes [80]. Because of these features, exosome-related miRNAs have been studied for early cancer diagnosis and treatment.

## 5 Exosomal microRNAs in cancer apoptosis

Tumor cell-released exosomes induce changes in recipient cells, promoting tumor growth, angiogenesis, stromal fibroblast activation, and alterations in cancer cell attachment. This facilitates premetastatic niche formation, suppresses host immune response, inhibits cell death, and leads to drug resistance. This section explores exomiRs' role in regulating apoptosis and therapy resistance (Table 1).

**Table 1:** Dysregulated exosomal miRNAs in cancer.

Disease	miRNA	Expression	Function	Ref
Lung Cancer	miR-7-5p	Downregulation	A tumor suppressor which inhibits proliferation, migration, and metastasis synergistically with Everolimus to induce apoptosis	[81]
	miR-613	Downregulation	A tumor suppressor which inhibits the growth and migration but induces cell apoptosis, exacerbates DNA damage, and reversal of chemoresistance	[82]
	miR-100-5p	Downregulation	Blocking mTOR expression to sensitize lung cancer cells to DDP	[83]
	miR-770	Downregulation	Suppression of MAP3K1 expression by inhibiting the polarization of M2 macrophages to preventing tumor growth and invasion	[84]
	miR-103a-3p	Upregulation	Originating from CAFs to promote of cisplatin resistance, and an inhibit apoptosis by targeting Bak1	[85]
	miR-126	Downregulation	Targeting ITGA6 to inhibit proliferation, cell cycle advancement, migration, and invasion, and indution of apoptosis	[86]
	miR-433	Downregulation	inhibits tumor growth, while promotes apoptosis, decrease drug resistance and infiltration of CD4 and CD8 T-cells by targeting TMED5	[87]
	miR-222-3p	Upregulation	Enhancing tumor cells proliferation, migration, invasion, anti-anoikis activity, and gemcitabine resistance by targeting SOCS3	[88]
	miR-338-3p	Downregulation	Inhibition of metastasis and induction of apoptosis by targeting CHL1	[89]
Colorectal Cancer	miR-92a-3p	Upregulation	Inhibiting FBXW7 and MOAP1 to enhance cell stemness, EMT, metastasis, and 5-FU/L-OHP resistance	[90]
	miR-96-5p and miR-149	Downregulation	Lower cell viability, higher cell apoptosis, and tumor growth inhibition	[91]
	miR-200c	Upregulation in serum/Downregulation in exosome	Inhibitory effects on metastasis through its ability to stimulate apoptosis in conjunction with LPS	[92]
	miRNA-129-5p	Downregulation	promoting apoptosis while proliferation, migration, and invasion are suppressed	[93]
Breast Cancer	miR-92	Upregulation	Impairment of apoptosis and proliferation of T-cells and enhancing tumor cell migration and proliferation	[94]
	miRNA-205	Upregulation	Promotes proliferation, migration, invasion, and tamoxifen resistance while preventing apoptosis in recipient BCCs by targeting E2F1	[95]
	miR-221/222	Upregulation	Suppressing P27 and Erα expression to promote tamoxifen resistance	[96]
	miR-9-5p	Upregulation	Increases drug resistance, inhibits apoptosis, and suppresses cell cycle arrest	[97]
Gastric Cancer	miR-21	Upregulation	Protects cells from chemotherapy-induced apoptosis by by regulating Bcl-2 and PTEN	[98]
	miR-15b-3p	Upregulation	Intensify tumor growth and malignant transformations and inhibit apoptosis through the DYNLT1/Caspase-3/Caspase-9 pathway	[99]
	miR-769-5p	Upregulation	Conferring DDP resistance and increases tumor progression by targeting CASP9 and inhibiting p53 ubiquitination	[100]
	miR-501	Upregulation	Fosters doxorubicin resistance and suppresses apoptosis by inactivating caspase-9/-3	[101]
Bladder Cancer	miR-148b-3p	Upregulation	Increases tumor proliferation, metastasis, and chemotherapy resistance but inhibits apoptosis by targeting PTEN, Bax and caspase-3	[102]
	miR-29c	Downregulation	Preventing cell proliferation and enhancing apoptosis	[103]
	miR-217	Upregulation	Releases by hBSC which promote tumor growth and migration while decreases apoptosis by modulating YAP	[104]
	miR-133b	Downregulation	Inhibits cell proliferation while promoting apoptosis by targeting DUSP1	[105]
	miR-139-5p	Downregulation	Suppression of PRC1 expression, which leads to the improvement of tumorigenic traits	[106]
Ovarian Cancer	miR-21	Upregulation	Increasing invasion and paclitaxel resistance and suppressing apoptosis by targeting APAF1 and Smad7	[107]
	miR-21-5p	Upregulation	increase in cell viability, glycolysis, and DDP resistance by targeting PDHA1	[108]
Pancreatic Cancer	miR-106b	Upregulation	Development of resistance to gemcitabine chemotherapy	[109]
	miR-1226-3P	Downregulation	Inhibiting cell proliferation and migration but induction of apoptosis	[110]
Prostate Cancer	miR-423-5p	Upregulation	Promotion of taxane resistance through inhibiting GREM2	[111]
	miR-145	Downregulation	Inhibits cell growth, promotes apoptosis, and reduces BclxL activity	[112]
Liver Cancer	miR-32-5p	Upregulation	Promoting angiogenesis and EMT, and emergence of multidrug resistance via PI3K/Akt pathway	[113]
	miR-638, miR-663a, miR-3648, and miR-4258	Upregulation	Promoting vascular permeability and initiating the pre-metastatic niche	[114]
Melanoma	miR-34a	Downregulation	Regulation of tumor cells sensitivity to cisplatin	[115]
	miR-125b-5p	Upregulation	inducing a tumor-promoting phenotype in macrophages by LIPA	[116]
	miR-494	Downregulation	induction of cell apoptosis by the downregulation of the Bcl-2	[117]

### 5.1 Lung cancer

In recent years, there has been a growing body of evidence supporting the notion that exosomal miRNAs, which can be transferred horizontally to recipient cells, play a crucial role in governing the intricate mechanisms underlying tumor progression and metastasis in this particular cancer type [118]. For instance, miR-7-5p is increasingly recognized as a crucial actor in tumorigenesis, exhibiting dual roles as both a promoter and inhibitor of tumor growth that shifts according to the cellular context. Comprehensive analysis, however, predominantly defines the primary function of miR-7-5p as a tumor suppressor, achieved through regulating multitudinous targets, including Akt, RelA, FAK, and KLF4 [[119], [120], [121], [122]]. Changes to the intracellular miRNA expression profile, including an increase in oncogenic miRNAs and a decrease in tumor-suppressing miRNAs, are brought about by mTOR inhibitors. This shift is accomplished by liberating miRNAs to the tumor microenvironment, effectively neutralizing the antitumor activity. One specific substance, Everolimus - a mTORC1 inhibitor and rapamycin derivative - reduces miR-7-5p levels within non-small-cell lung cancer (NSCLC) cells. This result is facilitated by exosome release carrying miR-7-5p and depends on the involvement of Rab27A and Rab27B. On the other hand, the activation of MNKs cultivates resistance to Everolimus through their persistent phosphorylation of eIF4E, a fundamental factor in building resistance to rapamycin. Reductions in intracellular miR-7-5p levels contribute to MNK/eIF4E pathway phosphorylation, thereby mitigating apoptosis. Conversely, combining miR-7-5p and Everolimus has demonstrated anticancer solid potency. This combination simultaneously inhibits the MNK/eIF4E and mTOR pathways, promoting apoptosis [81].

Current research implies that exosomes, originating from cisplatin (DDP)-resistant cells, hold a key position in the DDP-induced apoptosis. The downregulation of miR-613 in tumor cells promotes the development of resistance to chemotherapy. However, findings indicate that introducing exo-miR-613 or increasing endogenous levels of miR-613 through targeted modulation of GJA1, TBP, and EIF-4E shows the potential to restore sensitivity to cisplatin. This therapeutic approach effectively inhibits cell proliferation, enhances DNA damage, and promotes increased apoptotic rates both in vitro and in vivo [82]. Consistent with other studies, observations reveal a significant decrease in the expression of miR-100-5p in exosomes derived from DDP-resistant cells. These exosomes confer resistance to cisplatin in recipient cells by influencing and suppressing mTOR expression, thereby exerting their effect in vitro and in vivo [83].

MiR-770 plays a crucial role in suppressing tumor growth and metastasis in cancer. Research has specifically demonstrated a decrease in miR-770 expression in NSCLC. This reduction is closely linked to the inhibition of cell proliferation by promoting apoptosis and impeding the formation of metastatic characteristics. Notably, tumor cells release exosomes containing abundant miR-770, which exert a suppressive impact on M2 macrophage polarization. This effect effectively restricts the invasive behavior of NSCLC cells by targeting MAP3K1(89). Exosomes derived from cancer-associated fibroblasts (CAFs) have been found to exhibit increased levels of miR-103a-3p. These miR-103a-3p-containing exosomes contribute to drug resistance against DDP (cisplatin) and hinder apoptosis. The underlying mechanism involves the downregulation of Bak1 mRNA and protein levels within tumor tissues by miR-103a-3p. However, functional studies utilizing AMO-miR-103a-3p have shown that elevating Bak1 expression enhances sensitivity to cisplatin and promotes apoptosis in tumor cells. The interaction between miR-103a-3p and Pumilio2 (Pum2) facilitates the packaging of miR-103a-3p into exosomes derived from CAFs. Interestingly, transfection of si-Pum2 does not affect the expression of miR-103a-3p but inhibits its presence in exosomes derived from CAFs [85].

The importance of miR-126 in lung cancer has been consistently highlighted by numerous studies [123]. Specifically, miR-126-5p has been identified as a critical regulator that deactivates the VEGF-A/VEGFR-2/ERK signaling pathway in lung cancer cells. This deactivation induces pro-apoptotic effects and impedes the cells' metastasizing ability [124]. Supporting these findings, Chen et al. demonstrated that the overexpression of miR-126-3p, derived from bone marrow mesenchymal stem cells (BMSCs), effectively inhibits the invasive characteristics and induces apoptosis in NSCLC cells. This regulatory effect is accomplished through the targeting and negative regulation of PTPN9 [125]. Furthermore, miR-126 has emerged as a tumor suppressor with reduced expression levels detected in exosomes derived from the serum of NSCLC patients. Notably, these serum-derived exosomes promote metastatic potential by downregulating E-cadherin and upregulating N-cadherin and Vimentin. Exosomes loaded with miR-126 also play a crucial role in promoting tumor growth by facilitating cell proliferation and suppressing apoptosis. Interestingly, miR-126 can be transferred to NSCLC cells through serum-derived exosomes, resulting in the acquisition of malignant traits. This transfer mechanism involves the suppression of an oncogene called ITGA6, which leads to the attenuation of metastatic behavior and tumor growth inhibition [86].

Exosomal miR-433 has been identified as another crucial factor, as it exhibits lower levels in the plasma of chemotherapy-resistant NSCLC patients compared to patients with chemotherapy-sensitive NSCLC and individuals with normal serum. Exosomes carrying miR-433 have been shown to impede tumor growth by promoting apoptosis and the infiltration of CD4 and CD8 T-cells. This effect is mediated by targeting and suppression of TMED5 and WNT/β-catenin signaling pathway. In addition, miR-433 has been shown to regulate DNA damage, decrease drug resistance to DDP, and inversely correlate with large tumor sizes, distant metastasis, and advanced TNM stages in patients with NSCLC [87]. The contribution of exosomes to gemcitabine resistance in non-small cell lung cancer (NSCLC) remains unclear. However, studies have revealed that exosomes derived from gemcitabine-resistant cells exhibit upregulated levels of miR-222-3p. Surprisingly, these exosomes enriched with miR-222-3p have the remarkable ability to trigger a more aggressive behavior in recipient-sensitive cells when taken up through caveolin- and lipid raft-dependent endocytosis. They achieve this by targeting the SOCS3/Jak2/Stat3 and Bcl-2 signaling pathways [88]. Notably, the persistent downregulation of SOCS3 leads to the activation of STAT3. Consequently, the expression of growth regulatory proteins including Cyclin D1, c-Myc, c-Fos, as well as anti-apoptotic proteins such as Bcl-2, Mcl-1, and Bcl-XL, is increased. This dysregulation ultimately leads to hyperproliferation and inhibition of apoptosis [126].

**Fig. 3:**
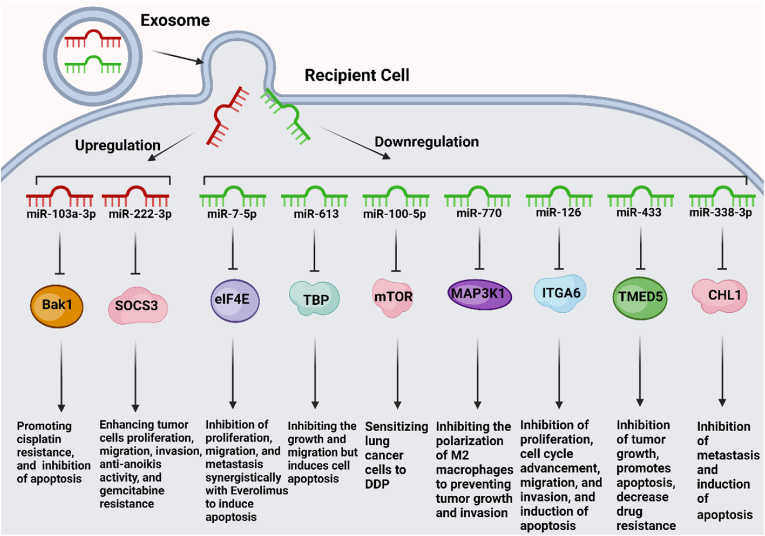
A diagram illustrating the role of Exosomal microRNAs and their intricate involvement in the signaling pathways associated with lung cancer. In this diagram, the diverse repertoire of Exosomal microRNAs implicated in lung cancer pathogenesis is depicted, highlighting their potential as diagnostic and therapeutic targets. Created with BioRender.com.

MiR-338-3p has also been identified as a significant contributor to lung tumorigenesis [[127], [128], [129], [130]]. It serves as a suppressive factor that hinders the transition to aggressive behavior in NSCLC cells by targeting an EMT-related factor called Sox4 [130]. A study by Tian et al. revealed that the expression levels of exosomal miR-338-3p were decreased in serum samples obtained from NSCLC patients. This reduction in exosomal miR-338-3p levels correlated with the induction of a metastatic phenotype in tumor cells. Additionally, the study demonstrated that the decreased levels of exosomal miR-338-3p led to increased expression of CHL1 through the activation of the MAPK signaling pathway. This, in turn, impeded the activation of pathways associated with apoptosis [89] (Fig. 3).

### 5.2 Colorectal cancer

The presence of miRNAs within exosomes has long been acknowledged for their pivotal involvement in regulating colorectal tumor growth. These miRNAs operate through various mechanisms, including the inhibition of apoptosis within tumor cells [131]. For example, exosomes released by CAFs within the tumor microenvironment have been observed to contain a high level of miR-92a-3p. The upregulation of miR-92a-3p results in the excessive activation of the Wnt/β-catenin pathway, which impairs mitochondrial apoptosis by directly inhibiting FBXW7 and MOAP1. This mechanism significantly contributes to the promotion of metastatic behavior and resistance to 5-fluorouracil/oxaliplatin (5-FU/L-OHP) treatment. Interestingly, the reintroduction of FBXW7 and MOAP1 can reverse the tumor-promoting effects observed in cells expressing miR-92a-3p. By promoting the ubiquitination degradation of β-catenin and inhibiting mitochondrial function, FBXW7 and MOAP1 restore the inhibition of cell stemness and promote cell apoptosis [90].

miR-34 and miR-145 are well-established anti-oncogenic miRNAs that exert regulatory control over various proto-oncogenes, thereby inhibiting the growth of colon cancer cells [132,133]. Specifically, miR-34a is crucial in overcoming resistance to 5-FU treatment by targeting Sirt1 and E2F3 [134]. In a study conducted by Akao et al., it was discovered that miR-145 and miR-34a are released through microvesicles (MVs). The levels of these miRNAs were significantly higher in MVs than in cells, and exposure to 5-FU influenced their levels in both cells and MVs. Intracellular levels of miR-145 and miR-34a increased in 5-FU-sensitive cells, disrupting the release of MV/miR-145, inhibiting cell growth and inducing tumor cell death. Conversely, the downregulation of miR-145 in 5-FU-resistant cells could be attributed to the enhanced release of both miR-145 and miR-34a through MVs following 5-FU exposure, suggesting a potential association between MV secretion and resistance to 5-FU [135].

Glypicans play a significant role in promoting organ development by modulating extracellular growth signals and forming morphogen gradients. However, in certain cancers, the expression of glypicans is upregulated, contributing to tumorigenesis, angiogenesis, and cancer progression and invasion [136]. A recent study has identified Glypican-1 (GPC1) positive exosomes as a specific CRC biomarker. The percentage of GPC1-positive exosomes in tumor tissues and plasma is significantly higher in CRC patients, approximately fourfold and over 10-fold, respectively. Furthermore, the study found that the loss of expression of miR-96-5p and miR-149 is observed in both tumor tissues and plasma of CRC patients, as well as in GPC1-positive exosomes derived from CRC tumor tissues and plasma, before surgical treatment. The levels of miR-96-5p and miR-149 in patients influence the expression of GPC1 protein and the secretion of GPC1-positive exosomes from tumor tissues. In vitro and in vivo experiments have demonstrated that restoring the expression levels of miR-96-5p and miR-149 inhibits GPC1 expression and the secretion of GPC1-positive exosomes, leading to reduced cell viability, increased cell apoptosis, and suppressed tumor growth. Additionally, two months following surgery, the plasma levels of GPC1-positive exosomes, GPC1 protein expression, miR-96-5p, and miR-149 return to normal levels [91].

Recent studies have indicated that lipopolysaccharide (LPS) can potentially impact the functions of exosomes by altering their composition [92]. Jiang et al. discovered that miR-200c levels were elevated in tumor tissues but reduced in serum exosomes, suggesting distinct roles for miR-200c in cellular and exosomal contexts. Stimulation with LPS enhanced miR-200c expression in exosomes, influencing the expression of Zinc finger E-box-binding homeobox-1 (ZEB-1) mRNA and its protein. Both miR-200c-3p in the cytoplasm and exosomes exhibited inhibitory effects on CRC migration and invasion by promoting apoptosis, particularly in conjunction with LPS, which is recognized as a natural inducer of apoptosis [92]. Functional experiments have explored the tumor-suppressive effects of miRNA-129-5p-loaded exosomes derived from colon cancer cells. These experiments aimed to investigate the capacity of these exosomes to induce apoptosis, as previously reported for miRNA-129-5p. Specifically, exosomes derived from tumor cells transfected with stable miRNA-129-5p exhibited selective apoptosis induction by decreasing Bcl-2 expression and increasing Bax expression. Additionally, these exosomes mitigated malignant characteristics associated with colon cancer [93].

### 5.3 Breast cancer

Extensive studies have elucidated discernible expression patterns of miRNAs in both normal and malignant breast cells [137]. In breast cancer cells (BCCs), exosomes derived from CAFs have been shown to induce the expression of PD-L1 and miR-92. miR-92 interacts with LATS2, a serine/threonine kinase, inhibiting its phosphorylation of YAP1. The decreased LATS2 expression leads to increased nuclear translocation of YAP1, resulting in the upregulation of PD-L1 expression by binding to its enhancer. The increased PD-L1 expression impairs apoptosis and proliferation of T-cells, thereby interfering with tumor-infiltrating immune cells and promoting tumor progression through miR-92 [94]. In recipient cells, exosomes derived from chemoresistance-resistant BCCs transfer miRNA-205, which can impact chemoresistance and tumorigenesis. miRNA-205 directly binds to the E2F Transcription Factor 1 (E2F1), activating the caspase pathway and phosphorylation of Akt. This leads to tamoxifen resistance and suppression of apoptosis in recipient BCCs [138]. E2F1 is a crucial cell cycle progression and apoptosis regulator in response to DNA damage [139]. Gain-of-function experiments have demonstrated that the resistant phenotype can be reversed by overexpressing E2F1 in BCCs [95].

Wei et al. found resistant cells released exosomal miR-221/222 into the tumor microenvironment, and miR-221/222 then entered recipient cells, which re-shape the microenvironment and increased Tamoxifen resistance. Enhanced communication and propagation of TAM resistance are facilitated by the presence of exosomal miR-221/222. Elevated levels of miR-221/222 hinder the expression of P27 and ERα, consequently fostering resistance to TAM in recipient cells. Consequently, recipient cells can be trained to become part of the “TAM resistance microenvironment" through this process [96]. Exosomes containing miR-9-5p, released by TAM-resistant breast cancer cells, have the ability to confer resistance to TAM-sensitive cells [96]. The presence of miR-9-5p negatively regulates ADIPOQ, a factor known for its tumor-limiting effects, thereby inhibiting cell apoptosis [[97], [140], [141]].

### 5.4 Gastric cancer

Tumor-associated macrophages (TAMs) are essential components of the tumor microenvironment, and they secrete various growth factors, cytokines, and molecules that regulate processes such as proliferation, invasion, metastasis, and angiogenesis [142,143]. Exosomes derived from M2 macrophages exhibit higher expression of miR-21 compared to inactivated macrophages. These exosomal miR-21 molecules can be transferred from macrophages to tumor cells, protecting against chemotherapy-induced apoptosis. This protection is achieved by regulating the expression of Bcl-2 and promoting the activation of the PI3K/AKT signaling pathways through the downregulation of PTEN [98]. In gastric cancer (GC), evidence suggests the involvement of an exo-miR-15b-3p/DYNLT1/Caspase-3/Caspase-9 regulatory network in the pathogenesis. miR-15b-3p is overexpressed in GC serum and cells, leading to increased expression of BCL-2 and decreased expression of BAX, cleaved caspase-9, and cleaved caspase-3. GC cells secrete exosomes containing miR-15b-3p, further enhancing tumor growth and malignant transformations and inhibiting apoptosis through the DYNLT1/Caspase-3/Caspase-9 pathway [99].

The aberrant expression of miR-769-5p is pivotal in the likelihood of malignant evolution across multiple tumors [[138], [139], [144], [145], [146]]. In patients with DDP resistance, miR-769-5p is increased in GC tissues and accumulates in serum exosomes. A miR-769-5p-loaded exosome confers resistance to DDP to sensitive cells by blocking apoptotic pathways by targeting CASP9 and inhibiting p53 ubiquitination via the RNF20‐NEDD4L‐p53 pathway, and miR‐769‐5p can suppress RNF20 expression. The RNF20 protein is vital in ubiquitinating the p53 protein, as it aids in directly degrading the p53 protein by the E3 ubiquitin ligase, NEDD4L [100]. Reports have suggested that miR-501 participates in the development of various cancer types, notably colorectal, gastric, cervical, and lung cancer [147]. In doxorubicin-resistant gastric cancer cells, exosomes (ADR Exos) exhibited overexpression of miR-501. These exosomes released by resistant cells enhance doxorubicin resistance in neighboring sensitive GC cells by suppressing apoptosis. This effect is achieved through the downregulation of BLID and subsequent inactivation of downstream caspase-9/-3. Moreover, the transfer of exosomal miR-501 promotes increased proliferation and invasion of recipient cells by inducing Akt phosphorylation after BLID downregulation. In vitro and in vivo studies have shown that knocking down miR-501 or overexpressing BLID in recipient cells counteracts ADR Exos's effects, reduces doxorubicin resistance, inhibits cell growth, and suppresses invasion. Notably, inhibiting exosome secretion in resistant cells has been found to inhibit Akt phosphorylation at Ser 473, further supporting the role of exosomes in this process [101].

### 5.5 Bladder cancer

Exosomal miRNAs derived from CAFs play a significant role in prostate tumorigenesis [148,149]. cancer cells can directly take up miR-148b-3p through exosomes produced by CAFs. This particular miRNA targets PTEN, a tumor-suppressor protein, and promotes cell proliferation, metastasis, and resistance to chemotherapy. It achieves this by blocking apoptotic pathways by downregulating Bax and caspase-3. Inhibition of miR-148b-3p or overexpression of PTEN in CAF-derived exosomes using loss-of-function experiments enhances chemosensitivity by deactivating the Wnt/β-catenin pathway [102]. The abnormal expression of the miRNA-29 family has been observed in various types of tumors, and these miRNAs can be transferred to neighboring cells through exosomes released by tumor cells. By introducing miRNA-29c into BIU-87 human bladder cancer cells via adenovirus transfection, the cells can release exosomes loaded with miRNA-29c. The miRNA-29c derived from exosomes induces apoptosis in tumor cells by downregulating BCL-2 and MCL-1 at both the mRNA and protein levels [103].

Exosomes derived from human normal bladder stromal cells (hBSCs) can influence the physiological conditions of recipient cells. Significant effects on bladder cancer proliferation, migration, and apoptosis have been observed through loss- and gain-of-function experiments using hBSC-derived exosomes containing either a miR-217 mimic or inhibitor. These effects are mediated by modulation of the transcription factor YAP and its target proteins, including Cyr61, CTGF, and ANKRD1. Treatment of cell lines with exosomal miR-217 mimic leads to increased expression of YAP and its target proteins, while treatment with exosomal miR-217 inhibitor results in a significant decrease in the expression of YAP and its target proteins [104]. Exosomes isolated from patient serum and bladder cancer tissues exhibit downregulation of miR-133b expression. Exosomal miR-133b functions by binding to the promoter region and promoting the upregulation of dual-specificity protein phosphatase 1 (DUSP1). This upregulation of DUSP1 leads to increased apoptosis. DUSP1 plays a crucial role in activating the JNK pathway and controlling apoptosis, cell cycle, and autophagy by dephosphorylating specific residues of MAPK subunits [105].

The levels of miR-139-5p, an important tumor suppressor, are significantly reduced in the cells and tissues of bladder cancer patients. Interestingly, exosomes derived from human umbilical cord mesenchymal stem cells (hUCMSCs) can transfer miR-139-5p to tumor cells, leading to the suppression of PRC1, a protein involved in tumorigenesis. This transfer of miR-139-5p results in the inhibition of cell proliferation through the downregulation of Bcl-2 and PCNA, as well as the upregulation of Bax. Furthermore, the migrative and invasive phenotype of tumor cells, along with EMT, is reversed upon silencing PRC1. This reversal is indicated by the dysregulation of EMT markers such as N-cadherin, Vimentin, and E-cadherin [106].

### 5.6 Ovarian cancer

Gynecological cancers are prevalent among women, and numerous studies have highlighted the significant role of exosomal miRNAs, particularly in cervical cancer [150,151]. Ovarian tumor-derived exosomes have been found to contain specific miRNA profiles [152,153]. The transfer of miR-21 through exosomes has been identified as a contributing factor to the metastatic behavior of tumor cells. Notably, miR-21 expression is higher in cancer-associated adipocytes (CAAs) and CAFs compared to malignant cells. This distinct expression pattern enables the transfer of miR-21 from CAAs or CAFs to neighboring cancer cells. As a result, these cancer cells become more resistant to chemotherapy, and their ability to undergo apoptosis when treated with paclitaxel is suppressed. These effects are achieved by downregulating APAF1, a crucial component in regulating apoptosis. Moreover, miR-21 indirectly affects MMP1 expression by targeting Smad7, a component of the TGF signaling pathway. The transfer of miR-21 to ovarian cancer cells via exosomes leads to an upregulation of MMP1 expression, resulting in increased invasion potential [107]. miR-21-5p has been implicated in various physiological and pathological processes. Consistent with previous studies, recent research revealed elevated levels of miR-21-5p in exosomes derived from ovarian tumor cells resistant to the chemotherapy drug DDP. The increased presence of miR-21-5p in exosomes hindered the sensitivity of ovarian cancer cells to DDP, leading to enhanced cell viability and increased glycolysis. Importantly, this study demonstrated that miR-21-5p targets the pyruvate dehydrogenase E1 subunit alpha 1 (PDHA1). The effects of miR-21-5p were counteracted by overexpressing PDHA1, confirming its direct involvement as a target molecule [108].

### 5.7 Pancreatic cancer

Pancreatic cancer (PC) exhibits the poorest survival rate compared to other types of cancer [154]. Analyzing distinct expression profiles of exosomal miRNAs can enhance our comprehension of the key driver genes in pancreatic cancer, as well as the downstream pathways involved. In various types of cancer, miR-106b is overexpressed, and it exhibits a significant function in the modulation of malignant phenotypes [155]. The latest research has shown that CAFs release exosomes that contain high levels of miRNA-106b, which can be taken up by pancreatic cancer cells in a manner that leads to resistance to gemcitabine chemotherapy. Furthermore, it has been observed that miRNA-106b directly targets TP53INP1 a tumor suppressor that plays a crucial role in regulating DNA repair responses in the face of cellular stress. By downregulating TP53INP1 through miRNA-106b, pancreatic cancer cells can withstand the effects of gemcitabine more effectively. Reversing gemcitabine resistance in pancreatic cancer cells holds promise through two potential strategies: knockdown of miRNA-106b or inhibition of exosome secretion from CAFs to pancreatic cancer cells [109]. Significant downregulation of exomiR-1226-3P was observed in patients with pancreatic ductal adenocarcinoma (PDAC) when compared to healthy controls. This decrease in exomiR-1226-3P levels was found to be associated with tumor invasiveness and metastatic behavior. On the other hand, overexpression of miRNA-1226-3P, achieved by suppressing MUC1, had contrasting effects on PDAC cells. It limited cell proliferation and migration, while also inducing apoptosis [110].

### 5.8 Prostate cancer

Dysregulation of exosome-derived miRNAs has been documented in the progression of prostate cancer, contributing to the reprogramming of target cells by influencing gene expression [156]. miR-423-5p has been found to play a crucial role in the resistance of prostate cancer cells to chemotherapy. Exosomes derived from CAFs exhibit increased expression of miR-423-5p, which has been implicated in promoting resistance to taxane-based chemotherapy. It achieves this by targeting and inhibiting GREM2 through the TGF-β pathway. Conversely, inhibiting TGF-β leads to a reduction in cell proliferation and an increase in apoptosis. These findings suggest that inhibiting TGF-β may be a potential strategy to counteract taxane resistance induced by CAF-produced exosomes in prostate cancer cells [111]. There is a growing body of evidence suggesting that adipose-derived stromal cells (ASCs) release exosomal miRNAs, specifically miR-145, in prostate cancer cells. In vitro experiments were conducted using a miR-145 hairpin inhibitor to suppress the expression of miR-145 in ASCs. The results demonstrated that miR-145 played a pivotal role in ASCs by inducing apoptosis and inhibiting the progression of prostate cancer cells. Furthermore, in vivo experiments showed that the local transplantation of ASCs effectively inhibited both metastasis and invasion of cancer cells primarily by promoting apoptosis [112].

### 5.9 Liver cancer

Emerging research has demonstrated that liver tumor cells have the ability to secrete exosomes containing functional effectors, such as miRNAs. These exosomal miRNAs exhibit multifaceted functions in the intricate regulation of apoptosis [157]. Studies have indicated that miR-32-5p plays a role in radioresistance, chemoresistance, and castration resistance in prostate cancer patients. However, its involvement in multidrug resistance in hepatocellular carcinoma (HCC) is still not well understood [158]. Exosomes derived from multidrug-resistant cells (Bel/5-FU) transfer miR-32-5p to sensitive cells, where it directly targets the PTEN gene. miR-32-5p has been found to activate the PI3K/Akt pathway, leading to angiogenesis, EMT, and multidrug resistance. Contrary to earlier reports, the study reveals that the activation of the PI3K/Akt pathway does not induce apoptosis in tumor cells [113]. The exosomes derived from highly metastatic HCC cells (HuH-7M) contain specific miRNAs (miR-638, miR-663a, miR-3648, and miR-4258) that promote vascular permeability and initiate the formation of the pre-metastatic niche. These miRNAs downregulate the expression of VE-cadherin and ZO-1, leading to increased proliferative ability and suppression of apoptosis [114].

### 5.10 Melanoma

Melanoma, regarded as the most lethal variant of skin cancer, represents a substantial proportion of mortality within the skin cancer category [159]. Notably, exosome-derived miRNAs have emerged as key contributors to the progression of melanoma, particularly by exerting a significant role in suppressing apoptosis [160]. For instance, miR-34a is regulated by p53 and is known for its ability to suppress tumors. There is speculation that miR-34a may play a role in cisplatin resistance, a common issue in cancer treatment. Interestingly, researchers have observed a gradual decrease in the expression of miR-34a in melanoma cells over time. Surprisingly, when these cells were treated with cisplatin, there was an increase in the release of miR-34a through exosomes, which made the cells insensitive to the drug. The study's findings demonstrated that increasing the expression of miR-34a improved the sensitivity of tumor cells to cisplatin. This was evident from the higher rate of apoptosis observed in vitro. By either introducing more miR-34a into the cells or using metformin to reduce the exosomal release induced by cisplatin, researchers were able to counteract this effect and enhance the sensitivity of tumor cells to cisplatin-induced cell death [115]. Exosomes derived from melanoma cells have a unique profile of miRNAs and can trigger a macrophage phenotype that promotes tumor growth. In a study by Gerloff et al., it was revealed that these melanoma exosomes carry a specific miRNA called miR-125b-5p, which induces a tumor-promoting phenotype in macrophages. By targeting lysosomal acid lipase, A (LIPA), miR-125b-5p promotes macrophage survival and prevents spontaneous apoptosis, thereby contributing to the development of tumor-associated macrophages. When macrophages take up melanoma exosomes, they acquire a hybrid phenotype that displays characteristics of both M1 and M2 macrophages. The transferred miR-125b-5p further enhances the activation of M1 macrophages, which aids in recruiting myeloid cells and promoting inflammation associated with cancer. Additionally, miR-125b-5p induces the expression of CD80, which potentially influences the specific immune response mediated by T cells [116]. miR-494 is found in large quantities in exosomes derived from melanoma cells and is detected at significantly higher levels in serum exosomal samples from melanoma patients compared to healthy individuals. Tumor cells have a mechanism for selectively sorting miR-494 into exosomes while reducing its expression level within the cells. This reduction of miR-494 promotes cancer progression. Functional studies have shown that increasing the expression of endogenous miR-494 or inhibiting exosome release by targeting rab27a can suppress the malignant characteristics of melanoma cells. This suppression occurs through the induction of cell apoptosis, which is facilitated by the downregulation of the Bcl-2 protein [117].

## 6 Advantages and application of exosomal miRNAs in cancer treatment

ExomiRs have demonstrated potential as a therapeutic approach to human cancer. They're involved in all kinds of cellular processes, stable under non-physiological conditions, and expressed specifically in tissues and disease states [[161], [162], [163], [164], [165]]. Additionally, tumorigenesis can be inhibited by eliminating exosomes from the circulatory system or preventing their uptake by target cells. It is possible to isolate exosomes from a patient's circulatory system, modify them, and relocate them to the same patient for cancer therapy [[166], [167], [168], [169]]. Unlike liposomes, exosomes have a longer half-life, so they bind to the receptors of specific cells more specifically [164]. There is evidence that exomiRs can resist freeze-thaw cycles, are stable during long-term storage at room temperature, and can be stored at −20 °C for up to five years [161,162]. Since exomiRs are more stable than proteins and other nucleic acids, they make excellent sampling and analysis agents [170]. Exosomes released from malignant tissue are significantly higher than those released from normal tissue. In addition, tumor liquid biopsy samples like plasma, urine, and ascites often exhibit elevated exomiR concentrations [[171], [172], [173]]. Exosomes with miRNAs loaded with tumor suppressors have been shown to suppress tumor angiogenesis when used against proangiogenic mRNAs. Specific disorders can also be treated with exosomes by delivering therapeutic genetic agents to target cells [174]. ExomiRs, being naturally occurring molecules, hold significant potential as nano-vectors for delivering targeted anticancer drugs with minimal immunogenicity. These unique characteristics help them escape undesirable immune reactions and inhibit tumor growth through gene knockout [175]. ExomiRs can cross the blood-brain barrier, escape endosomes, and exhibit low toxicity. Targeted delivery minimizes systemic toxicity due to their specific binding to tumor cells [176,177].

## 7 Challenges related to the utilization of exosomal miRNAs

**Fig. 4:**
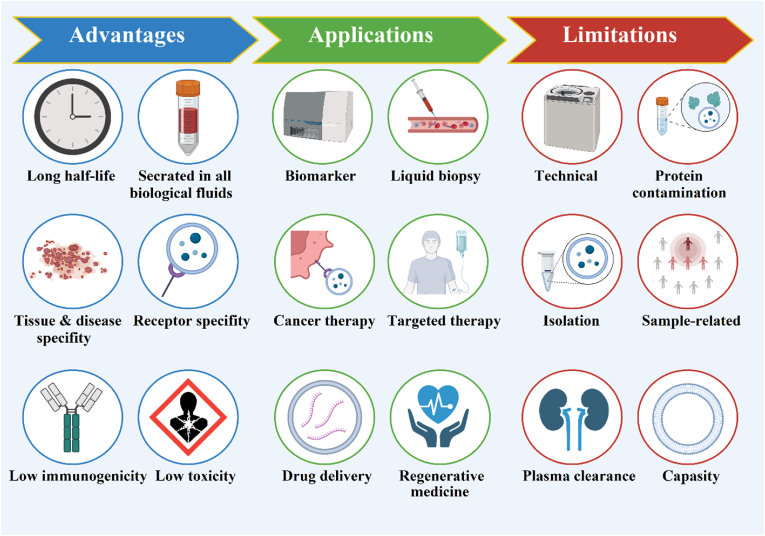
Three main aspects of using exosomes in biomedical research. Created with BioRender.com.

There are numerous benefits associated with the use of exomiRs in cancer management, but there are also several technical and scientific challenges. Currently, there is no widely accepted method for effectively isolating and purifying exomiRs, and the impact of different techniques on miRNA expression profiling is still under investigation. The isolation of individual extracellular vesicles (EVs) from complex bodily fluids requires the establishment of standardized methodologies. Additionally, the identification of well-characterized reference standards that are publicly accessible for the collection, storage, and processing of EV-containing body fluids remains challenging [[178], [179], [180]]. Furthermore, body fluids are a rich source of exosomes originating from tumors and normal cells, so it is difficult to isolate a tumor-derived exosome. During the separation process, protein fragments can be contained within urine and serum, which can interfere with the results [181,182]. Establishing an appropriate normalization procedure for exomiR expression levels using an endogenous housekeeping miRNA is yet to be accomplished. The identification of biases related to sample handling and their influence on exomiR expression poses an ongoing challenge. To obtain accurate results from small-sample analyses, large-scale prospective cohort studies are necessary. The rapid clearance of exomiRs from plasma is another significant challenge. After administration, exomiRs have a short half-life in the body because of enzymatic degradation and renal clearance. Their limited stability and persistence in the bloodstream hinder their effective delivery. Another challenge is exomiRs' limited drug loading capacity. ExomiRs are naturally occurring small RNA molecules, and their size constraints restrict their therapeutic cargo. Due to this limitation, exomiRs are unable to deliver sufficient quantities of therapeutic agents, such as small molecules or nucleic acid-based drugs. Also, exomiR properties can differ depending on the cell type. Various cells and tissues release exosomes with different cargo compositions, including different miRNA sets. In designing therapeutic strategies and interpreting experimental results, this heterogeneity adds an additional layer of complexity [[183], [184], [185], [186]]. Overcoming these obstacles requires further investigation into exosomes and their implications for diverse therapeutic approaches (Fig. 4) [187].

## 8 Conclusion and future prospective

Exosomes are critical in transferring miRNAs between cells, essential for intercellular communication and transportation. This mechanism contributes to cancer progression and resistance to apoptosis. The intricate interaction between exomiRs and apoptotic signaling pathways regulates cancer cell survival and tumor growth.

ExomiRs have emerged as promising diagnostic and prognostic biomarkers, although their clinical utility is still being explored. It has been found that there is dysregulation of specific exomiRs in different types of cancers, and their expression levels are associated with tumor progression, tumor stage, and treatment response. It has been studied that exosome can be isolated from biofluids, including blood, urine, and saliva. They have shown great promise as non-invasive biomarkers for cancer detection and monitoring in the future. Also, exomiRs can be used as therapeutic agents to enhance chemotherapy sensitivity in cancer cells. As a therapeutic agent for cancer treatment, exomiRs represent an exciting frontier in oncology research. Exosomes can naturally deliver bioactive molecules to recipient cells, which researchers can use to selectively target cancer cells and increase their sensitivity to conventional chemotherapeutics.

Although exomiR holds excellent promise as a diagnostic and therapeutic tool for cancer, its translation into clinical practice poses significant challenges. Standardizing methods for exosome isolation and quantification is essential, as is identifying suitable controls to normalize data and establish reliable and reproducible assays for clinical use. In conclusion, exomiR research is entering a new phase in cancer biology and therapy. miRNAs cargo carried by exosomes represents a promising avenue for understanding and treating cancer resistance to apoptosis. Continued research will fuel the development of new biomarkers and therapeutic targets. In the fight against cancer, exomiRs hold significant promise for improving patient outcomes.

## Funding

The authors declare that no funds, grants, or other support were received during the preparation of this manuscript.

## CRediT authorship contribution statement

**Mohammad Salehi:** Writing – review & editing, Writing – original draft, Visualization, Project administration, Methodology, Investigation, Conceptualization. **Mohammad Javad Kamali:** Writing – review & editing, Writing – original draft, Visualization, Methodology, Investigation, Conceptualization. **Daniyal Arab:** Writing – review & editing, Validation, Investigation. **Naghme Safaeian:** Writing – review & editing, Visualization, Methodology, Investigation. **Zahra Ashuori:** Writing – review & editing, Investigation. **Moein Maddahi:** Writing – review & editing, Investigation. **Narges Latifi:** Writing – review & editing, Methodology. **Amir Moein Jahromi:** Writing – original draft, Supervision, Conceptualization.

## Declaration of competing interest

The authors declare that they have no competing interests.
